# Plectin: Dual Participation in Tumor Progression

**DOI:** 10.3390/biom14091050

**Published:** 2024-08-24

**Authors:** Zhihui Wang, Wenbin Wang, Qing Luo, Guanbin Song

**Affiliations:** 1 College of Bioengineering, Chongqing University, Chongqing 400030, China; 202219021037@stu.cqu.edu.cn (Z.W.); 202319021096t@stu.cqu.edu.cn (W.W.); qing.luo@cqu.edu.cn (Q.L.); 2 Key Laboratory of Biorheological Science & Technology, Ministry of Education, Chongqing University, Chongqing 400030, China

**Keywords:** plectin, cytoskeleton, tumorigenesis, tumor development, biomolecules

## Abstract

The *plectin* gene can encode a cytoskeletal linking protein, plectin, known for its interaction with three critical components of the cellular cytoskeleton: intermediate filaments, microtubules, and actin filaments. In recent years, more and more studies have reported that plectin is closely related to tumorigenesis and development, exhibiting both tumor-suppressive and tumor-promoting functions. Here, we first introduce the molecular structure and function of plectin, and then we summarize the current understanding of the crucial role of plectin in cancer progression. Finally, we also discuss the possible reasons for the different roles of plectin expression in various types of cancer and highlight the double-edged sword role of plectin in tumor progression. The review aims to deepen the comprehensive understanding of plectin’s role in cancer and further help to develop novel therapeutic strategies and drug targets.

## 1. Introduction

Plectin, discovered about 40 years ago, plays an important role in the organization and function of the skeleton in vertebrate cells [1,2,3]. It is a large protein with a molecular weight of about 500 kDa and belongs to a family of proteins called plakin [4]. Plectin is vital for maintaining structural integrity within cells and plays a critical role in tissue/cell mechanics and mechanotransduction by cross-linking the cytoskeleton [5].

In the last few years, one of the most exciting advancements in the research on plectin is its emerging role as a potential driving factor in numerous human cancers [6,7,8,9,10,11,12,13,14]. Abnormal expression of plectin has often been found in various tumor types [15] The coordination of the cytoskeletal network and its dynamics is a fundamental characteristic of cell migration and cancer cell invasion [16]. Plectin actively participates in numerous cellular activities that contribute to tumor initiation and progression, including cell proliferation, adhesion, migration, invasion, and signal transduction [3]. These activities are intricately linked to dynamic changes in the cytoskeleton and adhesion processes, ultimately facilitating cellular invasion and metastasis. The dynamic interplay between plectin and these cytoskeletal components underscores its pivotal role in cancer cells and tumor tissue [8].

Given the diverse potential interactions and strategic significance of plectin, it is not surprising that its functional impairment can lead to various diseases and disorders [17]. In fact, plectin has emerged as a prototypical protein, and its functional disruptions are implicated in multisystemic disorders, affecting different tissues, cell types, and organs. The importance of plectin in cancer cells and tumor tissue has been confirmed in recent years [18,19,20]. Therefore, in-depth exploration of the role and regulatory mechanisms of plectin in cancer is of paramount significance. In this review, we focus on the crucial role of plectin and provide a deeper understanding of its involvement in cancer progression.

## 2. Molecular Structure and Function of Plectin

**Figure 1:**
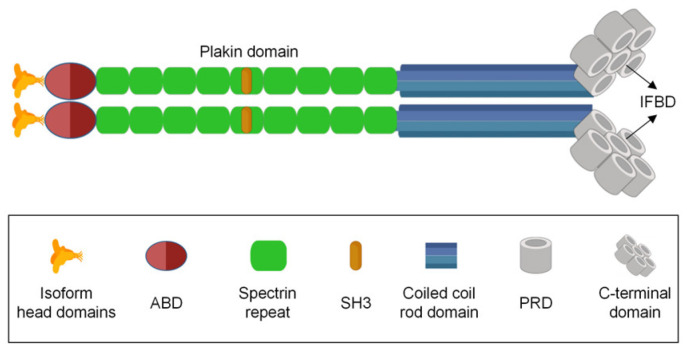
Schematic representation of the domain organization of plectin. Plectin is a dimer; each monomer consists of an actin-binding domain (ABD), spectrin repeat, rod domain, and plectin repeat domain (PRD). The orange regions at the N-termini of the polypeptide chains represent isoform-specific head domains. Within the N-terminal domain, there are two ABDs, each composed of two calponin homology domains (light and dark red). There are two plakin domains, each consisting of nine spectrin repeats (green) and one Src homology-3 (SH3) domain (brown). The central coiled-coil rod domain spans a length of 200 nm. Moving toward the C-terminal domains, each contains six PRDs. These PRDs comprise a conserved region, referred to as a module, and a linker region. One of the linker regions houses the universal intermediate filament-binding domain (IFBD).

*Plectin* is a fascinating gene with various functions. It is encoded by a single gene located on chromosome 8 in humans [21,22] and on chromosome 15 in mice [23,24]. Plectin belongs to the plakin family, which helps connect the cytoskeleton. Due to its high molecular weight and multidomain structure, plectin has drawn widespread attention. It is a dimeric protein, with each monomer consisting of specific structural components. The polypeptide chain of plectin comprises approximately 4500 amino acids, including a central α-helical coiled-coil rod domain, C-terminal domains, and N-terminal globular domains (as shown in Figure 1). The N-terminal domain undergoes variable splicing, giving rise to 12 isoforms of plectin (plectin1, plectin 1a–1k) [25,26].

Plectin plays a vital role in providing structural support within the cell [27,28]. It was initially identified as a primary component of intermediate filaments [29]. The actin-binding domain (ABD) interacts with actin, while the plakin domain enables lateral connections with various cytoskeletal filaments, such as microtubules, intermediate filaments, and microfilaments, forming a dense structural support system. Additionally, plectin can bind to cell adhesion molecules, including integrins and actin-binding proteins. This multidomain structure of plectin, with its dynamic spatiotemporal regulation through alternative splicing, underscores its significance in maintaining cell integrity, cytoskeletal organization, and participation in various cellular processes [30]. The versatility of plectin’s structure and function positions it as a key player in the intricate network of cellular architecture and signaling [3]. Currently, plectin is recognized as one of the most multifunctional cytoskeletal linker proteins, playing a crucial role in regulating cellular movement, particularly in processes such as cell migration and muscle contraction [31]. Primarily localized in the cytoplasm, plectin engages in intricate interactions with various cytoskeletal proteins and signaling molecules.

Furthermore, plectin binds to cell adhesion molecules presented on these filamentous structures, establishing a cohesive network that significantly contributes to cellular integrity and functionality. The ABD in plectin can interact with integrin α6β4 [32], nesprin-3α [33,34,35], and anti-muscle atrophy protein [36]. These interactions, in conjunction with plectin acting as a “recruiter,” are crucial for resisting the contraction and traction forces generated by actin [37]. Following ABD, there is a plakin domain consisting of nine spectrin repeat sequences, which binds to integrin β4 [38]. Each spectrin repeat sequence is composed of three α-helices, forming anti-parallel triple-helix bundles [39]. The plakin domain also interacts with intermediate filaments, with each domain exhibiting specific affinities for different types of intermediate filaments [1,26]. Plectin maintains the structural integrity of cells by connecting and organizing various components of the cytoskeleton.

In addition, plectin is implicated in cell signaling pathways, participating in the regulation of cellular physiological activities. The structure of plectin includes the sarcoma gene (Src) homology-3 (SH3) domain, which may interact with signaling molecules such as Src kinase. Src kinase is a crucial protein kinase that plays a key role in cell signaling involved in multiple biological processes, including cell proliferation, migration, and survival [40]. Studies have indicated that the simultaneous loss of phosphatase and tensin homolog (PTEN) and hemidesmosomal adhesions results in various tumorigenic properties, including proliferation, migration, resistance to anoikis, apoptosis, drug resistance, and increased metastatic capacity. Interestingly, these effects depend on plectin. Moreover, in PTEN-negative prostate cancer cells, plectin binds to actin-rich adhesions, leading to the activation of epidermal growth factor receptor (EGFR)/phosphoinositide 3-kinase (PI3K)/protein kinase B (PKB) and focal adhesion kinase (FAK)/Src pathways [41,42,43].

Plectin, through alternative splicing, generates a variety of isoforms that play a critical role in maintaining the integrity and function of cellular structures [2,15]. In normal tissues, these plectin isoforms have specific localizations and functions: In the skin, plectin 1a and plectin 1d are primarily involved in the construction of the intermediate filament cytoskeleton, ensuring the structural stability of skin cells [17,23,44]. In cardiac tissue, the presence of plectin 1a, plectin 1d, and plectin 1f is crucial for intercellular connections and the interaction of cells with the extracellular matrix [17,23,44]. In skeletal muscle, plectin 1, plectin 1b, plectin 1d, and plectin 1f anchor intermediate filaments to the nuclear/endoplasmic reticulum membrane system, mitochondria, Z-lines, and focal adhesions, playing a decisive role in maintaining the integrity of muscle fibers [18,23,24,44]. In cancerous tissues, there are significant differences in the expression levels of plectin isoforms, which may play a role in the development and progression of cancer. For instance, in non-small-cell lung cancer (NSCLC) cell lines, plectin1a and plectin1f are highly expressed, while they are not expressed in small-cell lung cancer (SCLC) cell lines [10,45]. Plectin1d may play a key role in the metastatic processes of various cancers, including breast cancer, kidney renal clear cell carcinoma (KIRC), kidney renal papillary carcinoma (KIRP), stomach adenocarcinoma (STAD), and thyroid carcinoma (THCA) [45].

Besides the critical role of plectin in cellular physiological processes, an even more important role of plectin in pathological process, especially in tumor initiation and progression, has been identified in recent years. Up to now, plectin has been found to be abnormally expressed in various types of tumors, indicating a potential key role of plectin in tumor onset and progression. Next, we focus on the current understanding of the role of plectin as well as how the plectin’s abnormal expression influences the progression of several tumors.

## 3. The Role of Plectin in Tumor Initiation and Progression

### 3.1. Plectin Promotes Cancer Development

This study found that plectin is significantly overexpressed in head and neck squamous-cell carcinoma (HNSCC) tissues, compared with noncancerous tissues. Patients with elevated plectin levels tend to have higher recurrence rates, leading to poorer prognoses and significantly reduced survival rates. Functional studies indicated that decreased plectin expression may inhibit the proliferation, migration, and invasion of HNSCC cells, possibly through the downregulation of extracellular signal-regulated kinases 1/2 (Erk 1/2) activity [8].

Likewise, analysis of the proteome in laser-captured microdissected tissue samples revealed elevated plectin expression in oral squamous-cell carcinoma (OSCC) compared with healthy oral mucosas [46]. Compared with normal epithelial cells, the expression level of plectin is further elevated in OSCC tumor tissues [47]. Other studies have also revealed strong and predominantly membranous plectin staining in OSCC tissues, whereas normal tongue mucosa exhibited only faint staining [48]. An absence of plectin in OSCC-derived cells results in decreased cell migration, invasion, and tumorigenic potential. Proposed mechanisms include reduced expression of actin-related proteins 2/3 (Arp 2/3) and decreased matrix metalloproteinase-9 (MMP-9) activity [49,50]. Additionally, the depletion of vimentin in OSCC-derived cells resulted in an augmented interaction with integrin β4 [51], suggesting that high levels of plectin expression in OSCC may be associated with tumor development.

Similarly, analysis of samples from colorectal cancer patients demonstrated a heightened plectin expression in adenocarcinomas and locally invasive nests relative to normal tissues [52]. Moreover, plectin expression is upregulated in SW480 colon cancer cells, and silencing plectin weakens the migration, invasion, and adhesive capabilities of SW480 cells, implying that plectin participates in actin assembly and invasion in colorectal cancer development processes [16]. Plectin functions as both an actin-binding protein and a scaffold for protein kinases, potentially contributing to the formation and stabilization of cellular adhesions. During the process of cancer cell invasion, the dynamic changes in actin are crucial for cell migration [53,54,55]. The regulatory effect of plectin on the structure of actin suggests that their interplay may offer novel molecular targets for the modulation of cell migration and invasion [16].

Furthermore, quantitative proteomic analysis identified plectin as a potential biomarker in esophageal squamous-cell carcinoma (ESCC), emphasizing its crucial role in this cancer development [56]. Plectin was reported to be upregulated two-fold in ESCC tissues. Immunohistochemical assays showed that plectin was overexpressed in ESCC patients, and most of them had cytoplasmic and membrane expression [56]. Nevertheless, the specific molecular mechanisms of plectin’s contribution to the onset and progression of ESCC are yet to be fully elucidated.

Elevated plectin levels are associated with both localized and metastatic prostate cancer (PCa), as opposed to benign tissues. Plectin knockdown inhibits cancer cell growth, colony formation, and xenograft growth. Proteomic analysis suggested that plectin regulates extracellular matrix, laminin, amino acid metabolism, cytoskeletal proteins, and cellular stress response, positioning it as a critical regulator of prostate cancer growth and metastasis [6]. It has been reported that one of the most frequent events during PCa pathogenesis is the loss of hemidesmosomes (HDs) [41,57,58]. The breakdown of HDs results in the release of integrin α6 and plectin, thereby promoting cell growth and migration in Pca [41]. In PTEN-negative prostate cancer cells, the disassembly of HDs leads to the association of plectin with actin-rich focal adhesions, which results in the activation of the EGFR/PI3K/Akt and FAK/Src pathways to promote tumor progression [41]. However, in PTEN-positive prostate and breast cancer cells, loss of integrin α6 or integrin β4 expression leads to a significant decrease in other heterodimeric partners and plectin, while in PTEN-negative cells, the protein levels of heterodimeric partners and plectin are only moderately affected or even upregulated. This suggests that the expression levels of plectin may be associated with the positive or negative status of PTEN in tumor cells [41,59].

Moreover, compared with normal liver tissues and cells, plectin expression is significantly increased in hepatocellular carcinoma (HCC) tissues and cells [60]. The knockdown of plectin leads to a marked decrease in the migration ability and epithelial–mesenchymal transition (EMT) of HCC cells compared with the control group. Further study revealed that plectin knockdown in HCC cells suppresses ERK1/2 phosphorylation [60]. These findings suggest that plectin may be a new prognostic indicator and potential target for HCC therapy.

Also, the research has demonstrated that plectin is a potential biomarker for intraductal papillary mucinous neoplasms (IPMN) of the pancreas, facilitating the differentiation between benign and malignant IPMN [61]. In this study, plectin expression was significantly higher in malignant IPMN when compared with benign IPMN. The expression of plectin commences in the early stages of carcinogenesis in IPMN, which may aid in the early detection of malignant tumors. Furthermore, plectin expression was not only observed in malignant IPMN but also elevated in lymph node metastases originating from malignant IPMN, assisting in the identification of metastatic tumors. Overall, the identification and detection of plectin can improve the efficiency of IPMN diagnosis and treatment [62].

Meanwhile, plectin also exhibits high expression in other tumors such as pancreatic ductal adenocarcinomas (PDACs), melanoma, bladder cancer, ovarian cancer, and endometrial cancer, indicating its tumorigenic role [11,63,64,65,66,67,68]. An increase in plectin expression is observed in PDACs, increasing with pancreatic carcinogenesis and retaining in metastatic foci [11]. In cases where clinical signs and symptoms cannot distinguish between PDAC and chronic pancreatitis (CP), the differential expression of plectin in malignant and benign tissues can aid in the differential diagnosis [69]. In melanoma, inhibition of plectin leads to the formation of low-density tumors, affecting tumor cell proliferation and adhesion processes [63]. In bladder cancer, plectin is closely associated with the formation of invadopodia and the cancer cell invasion. Plectin facilitates and stabilizes the function of invadopodia by forming connections with vimentin [64]. Therefore, it is considered a key molecule to regulate the invasion and metastasis of bladder cancer.

Currently, a number of studies have indicated that plectin expression notably increases and promotes tumor onset and progression in several tumors, as shown in Table 1.

### 3.2. Plectin Inhibits Cancer Development

In the recent two decades, the general role of plectin in cancer has been recognized. Although extensive research has revealed that increased expression levels of plectin can promote the occurrence and progression of certain types of cancer, conversely, a series of studies have also indicated that decreased expression levels of plectin can similarly drive the development of a variety of tumors. Moving forward, we review the main progress of this, as shown in Table 2.

As we discussed earlier, research has shown that plectin expression is significantly increased in HCC tissues and cells compared with normal liver tissues and cells. However, other studies have also shown significantly reduced plectin expression in PLC-PRF-5 and HepG2 HCC cells compared with Chang liver cells [12,13,72,73,74]. Plectin downregulation correlates with elevated E-cadherin levels, enhancing cell motility and collective migration [75]. The examination of HCC cases reveals a lack of plectin expression, potentially attributed to post-translational modifications [12]. Intermediate filament extracts from liver and hepatoma tissues exhibited irregular bundling of keratin fibers, suggesting that plectin deficiency impacts filament bundle integrity [12]. Hepatic cells lacking plectin show enhanced motility, akin to invasive behavior seen in HCC cells [75]. In addition, plectin deficiency in HCC leads to increased FAK and Rac1-GTPase activity, enhancing cell motility and migration [13,76]. Studies have shown that plectin protects podocytes from ADR-induced cell apoptosis and F-actin cytoskeleton disruption by inhibiting integrin α6β4/FAK/p38 pathway activation and plays an important role in the phosphorylation of FAK [13,77]. Moreover, downregulation of plectin increases Rac1 activity, thereby promoting tumor cell migration [13,78].

During early skin tumor development, integrin α6β4 recruits plectin to the plasma membrane, exerting an inhibitory effect. In basal-cell carcinoma (BCC), plectin expression is significantly reduced compared with that of normal skin, while squamous-cell carcinoma (SCC) and in situ carcinoma show a mild decrease [14]. It has been reported that plectin, in conjunction with bullous pemphigoid antigen 1e (BPAG1e), anchors intermediate filaments to HDs, preserving the integrity of these structures. Dissociation or depletion of plectin can destabilize HDs, altering their structure and impairing cell adhesion to the basement membrane [19,79]. In BCC, the significant reduction in plectin may lead to improper synthesis or assembly of HDs’ anchor filament complexes, potentially contributing to alterations in adhesive structures around the tumor periphery [15,80]. Moreover, it has been demonstrated that in p53 and Smad4 double-deficient mouse skin tumor-initiating cells (mTICs), integrin α6β4 recruits plectin to the cell membrane, which contributes to tumor suppression [76].

The interaction between plectin and breast cancer susceptibility gene 2 (BRCA2) affects centrosome localization, and correct localization plays a significant role in preventing genomic instability and cancer development. Inhibition of this interaction can lead to centrosome detachment and an increased rate of micronuclei formation, thereby potentially facilitating the onset of cancer [71].

From these findings, together with the aforementioned results, we can see that the effect of plectin in cancer onset and progression is quite complex, which presents a dual action. On the one hand, plectin expression obviously increases in some types of tumors and promotes tumor progression, such as HNSCC, OSCC, colon cancer, ESCC, and so on (Table 1). On the other hand, low expression of plectin promotes progression in several tumors, including BCC, SCC, and breast cancer (Table 2). More importantly, even if in the same type of tumor (e.g., HCC), while studies have found that high expression of plectin promotes tumor progression, other studies have also reported an inhibitory effect of the high expression of plectin in HCC cells. The integration of these discoveries underscores the intricate role of plectin in cancer initiation and progression, which acts as a double-edged sword.

Next, we discuss the possible reasons for the dual role. First, the association of plectin expression with overall survival varies across multiple cancers. A high mRNA expression of plectin is significantly correlated with poorer overall survival in several cancers, including PDACs, lung adenocarcinoma, and HNSCC. These cancers exhibit confirmed pro-tumorigenic regulatory factors [8,9,10]. Conversely, the low expression of plectin is considered an indicator of poorer overall survival in sarcoma, thymoma, pheochromocytoma, and paraganglioma, suggesting that the role of plectin in cancer may depend on tissue- or context-specific factors [15]. Second, research has reported that integrin α6β4 plays a dual role in epidermal tumors, either inhibiting or promoting cancer, and this is associated with plectin [76]. Initially, plectin’s recruitment to the plasma membrane by integrin α6β4 mediates tumor suppression [76]. However, later in tumor development, the presence of oncogenes such as Ras can shift plectin’s role to one of promoting tumor growth through the Erk pathway [76,81]. Considering the dual role of integrin α6β4 in cancer progression and its interaction with plectin, we speculate that the dual functionality of plectin may stem from the association between them. Future research is needed to further confirm this hypothesis and provide more in-depth evidence.

In summary, the dual effects of plectin in tumor progression may be subject to the integrated regulation of various factors, including cancer type, tissue specificity, and interactions with integrin α6β4. Further investigation into the precise functions and mechanisms of plectin across various cancers is essential for deepening our comprehension and may lead to the identification of novel therapeutic targets.

## 4. Summary and Perspective

**Figure 2:**
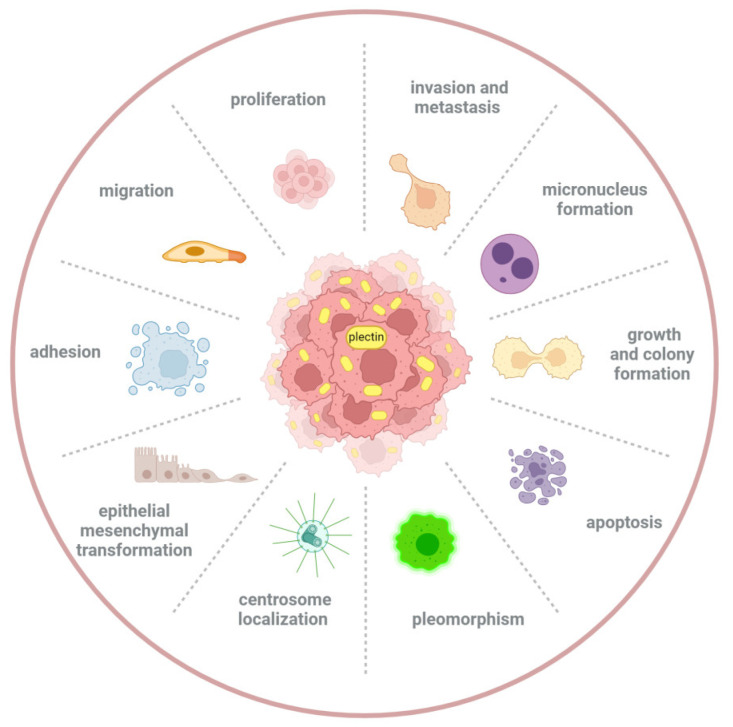
Several important biological behaviors affected by plectin in cancer cells. As a critical structural protein and cytoskeletal cross-linker, plectin can regulate various biological behaviors of cancer cells, not only including growth, proliferation, adhesion, migration, and invasion but also apoptosis, EMT, pleomorphism, and even micronucleus formation and centrosome localization, which takes a significant role in the occurrence and development of cancers.

As a well-established key biomolecule and regulator in the stability of cells and tissues, plectin plays a crucial role in maintaining the integrity of cellular structure and facilitating signal transduction. In particular, a growing body of evidence reports the key role of plectin in the initiation and progression of cancer, which has increasingly aroused extensive attention in the past dozen years. Tumor progression is a multistage complex process involving a multitude of biological changes, including cell cycle dysregulation, excessive proliferation, basement membrane disruption, EMT, extracellular matrix (ECM) stiffening, angiogenesis, local invasion, and distant metastasis as key steps [82,83,84,85,86,87]. Throughout the multifaceted stages of tumor progression, plectin has emerged as a pivotal regulator of cellular behavior, not only including growth, proliferation, adhesion, migration, and invasion, but also apoptosis, EMT, pleomorphism, and even micronucleus formation and centrosome localization (Figure 2) [6,43,45,71,88,89,90,91,92].

Along with the thorough research on plectin, our understanding of its important roles in cancer has largely increased; however, there are still some questions in this field to be answered. Firstly, although existing studies have confirmed that abnormal expression of plectin can affect key biological processes, including the proliferation, migration, invasion, and apoptosis of cancer cells, the specific molecular mechanisms of its action during various stages of different cancers remain unclear. In particular, the relationship between plectin and adhesion molecules is closely intertwined. In the progression of cancer, how changes in plectin affect adhesion molecules and the molecular mechanisms underlying these alterations are key to advancing our research horizons. Notably, the connection between plectin and fundamental biological processes in cancer progression, including cell cycle dysregulation, basement membrane disruption, and angiogenesis, requires definitive evidence. Addressing these gaps is crucial for advancing our understanding and for future investigative endeavors in oncology.

Secondly, while a wealth of experimental evidence demonstrates the dual action of plectin in the occurrence and development of cancers, the reason for this result remains unclear to date. Does it depend on the various types of cancer, the stages of cancer development, or the diverse differentiation status of cancer cells? Could this dual action be potentially related to the functions of other molecules that can interact with plectin, such as keratins, collagens, SNRPA1, and Fer? Is this dual function caused by the isoform variation of plectin? The details of this result have not been fully investigated.

Thirdly, a multitude of research literature has confirmed that the inhibition of plectin significantly affects a variety of cancer-related biological behaviors, highlighting the important role of plectin expression level changes in the occurrence and progression of cancer. At the same time, the latest research also points out that the repositioning of plectin within the cell can also affect the development of cancer. For example, it is reported that plectin is recruited to the cell membrane from cytoplasm in mouse skin tumor–initiating cells, while no change is observed in plectin expression [76]. This raises a key question: in the process of cancer, is it the change in the expression level of plectin or the change in its positioning within the cell that plays a more critical role? Future research needs to delve into the specific impact of these two changes in plectin on the process of cancer to fully understand its role in tumor biology. Moreover, this is a very interesting phenomenon in plectin localization dynamics, and it would be very worthwhile to explore the role of subcellular translocation of plectin within cancer cells, which would provide a deeper understanding of the regulatory mechanisms of plectin in cancer cell adhesion, migration, invasion, and metastasis.

Fourthly, as a cytoskeletal cross-linker, plectin acts as a crucial regulator in maintaining cellular tensional homeostasis and plays a critical role in tissue/cell mechanics and mechanotransduction. It is noteworthy that most solid tumor tissue/cells exhibit significant changes in mechanical properties, and the important roles of mechanical signals have also been confirmed in the occurrence and progression of cancer in recent decades [93,94,95,96,97]. Given the pivotal role of plectin in tissue/cell tensional homeostasis and mechanics, it is very necessary to further explore whether plectin acts as a mechanosensor and what the underlying mechanisms (i.e., mechanotransduction) are during cancer progression. However, these issues remain largely unaddressed so far. Addressing these questions will significantly enhance our comprehensive understanding of the multifaceted roles plectin plays in tumor progression, as well as opening up new avenues for studying and treating cancer based on plectin.

## Figures and Tables

**Table 1:** High expression of plectin promotes progression in several tumors.

Tumor Types	Outcomes	Signal Molecules	Refs.
HNSCC	Increased proliferation, migration, and invasion	Erk1/2	[8]
OSCC	Increased migration, invasion, tumorigenicity and levels of F-actin	Integrin β4, Cdc42, F-actin, Arp 2/3, MMP-9, NDRG1	[46,49]
Colon cancer	Increased migration, invasion and adhesion	Actin	[16]
ESCC	Promotes the occurrence of ESCC	Caspase-8	[56]
IPMN	Enhances the malignance	Unknown	[61]
PDAC	Increased migration and invasion; a specific marker	BRCA2	[11]
Prostate cancer	Increased growth, metastasis, invasion and colony formation	Clusterin, NNMT, QARS, RPS2, RPLP0, GRHPR, GlnRS, Actin, Glutamine	[6]
HCC	Increased migration, invasion and EMT	Erk1/2	[60]
Melanoma	Increased proliferation, Src activity and cell adhesion	Src	[63]
Bladder cancer	Increased migration, invasion and metastases	Vimentin, Cortactin, F-actin, MMPs	[64]
Lung adenocarcinoma	Increased migration and invasion	Erk1/2	[70]
Ovarian cancer	Increased invasion	Actin	[68]

Arp 2/3: actin-related proteins 2/3; BRCA2: breast cancer 2; Cdc42: cell division control protein 42; EMT: epithelial–mesenchymal transformation; ERK1/2: extracellular regulated protein kinases 1/2; ESCC: esophageal squamous-cell carcinoma; GlnRS: Glutamine tRNA ligase; GRHPR: Glyoxylate and hydroxypyruvate reductase; HCC: hepatocellular carcinoma; HNSCC: head and neck squamous-cell carcinoma; IPMN: intraductal papillary mucinous neoplasms; MMP-9: matrix metalloproteinase-9; MMPs: matrix metalloproteinases; NDRG1: N-myc downstream-regulated gene 1; NNMT: Nicotinamide N-methyltransferase; OSCC: oral squamous-cell carcinoma; PDAC: pancreatic ductal adenocarcinoma; QARS: Glutaminyl-tRNA synthetase; RPLP0: ribosomal protein lateral stalk subunit P0; RPS2: ribosomal protein S2; Src: sarcoma gene.

**Table 2:** Low expression of plectin promotes progression in several tumors.

Tumor types	Outcomes	Signal Molecules	Refs.
HCC	Increases cell motility and causes pleomorphism of cancer cells	FAK, Rac1-GTPase	[12,13]
BCC	Increases invasion and metastasis	Integrin α6β4	[14]
SCC			
Situ skin carcinomas			
Breast cancer	Promotes nuclear centrosome dissociation and micronucleus formation	BRCA2, centrosome	[71]

BCC: basal-cell carcinoma; BRCA2: breast cancer 2; FAK: focal adhesion kinase; HCC: hepatocellular carcinoma; SCC: squamous-cell carcinoma.

## References

[1] Wiche G. (1998). Role of plectin in cytoskeleton organization and dynamics. J. Cell Sci..

[2] Wiche G., Winter L. (2011). Plectin isoforms as organizers of intermediate filament cytoarchitecture. Bioarchitecture.

[3] Castañón M.J., Walko G., Winter L., Wiche G. (2013). Plectin-intermediate filament partnership in skin, skeletal muscle, and peripheral nerve. Histochem. Cell Biol..

[4] Walko G., Castañón M.J., Wiche G. (2015). Molecular architecture and function of the hemidesmosome. Cell Tissue Res..

[5] Wiche G., Osmanagic-Myers S., Castañón M.J. (2015). Networking and anchoring through plectin: A key to IF functionality and mechanotransduction. Curr. Opin. Cell Biol..

[6] Buckup M., Rice M.A., Hsu E.C., Garcia-Marques F., Liu S., Aslan M., Bermudez A., Huang J., Pitteri S.J., Stoyanova T. (2021). Plectin is a regulator of prostate cancer growth and metastasis. Oncogene.

[7] Gao K., Gao Z., Xia M., Li H., Di J. (2023). Role of plectin and its interacting molecules in cancer. Med. Oncol..

[8] Katada K., Tomonaga T., Satoh M., Matsushita K., Tonoike Y., Kodera Y., Hanazawa T., Nomura F., Okamoto Y. (2012). Plectin promotes migration and invasion of cancer cells and is a novel prognostic marker for head and neck squamous cell carcinoma. J. Proteom..

[9] Shin S.J., Smith J.A., Rezniczek G.A., Pan S., Chen R., Brentnall T.A., Wiche G., Kelly K.A. (2013). Unexpected gain of function for the scaffolding protein plectin due to mislocalization in pancreatic cancer. Proc. Natl. Acad. Sci. USA.

[10] Raymond A.C., Gao B., Girard L., Minna J.D., Gomika Udugamasooriya D. (2019). Unbiased peptoid combinatorial cell screen identifies plectin protein as a potential biomarker for lung cancer stem cells. Sci. Rep..

[11] Bausch D., Thomas S., Mino-Kenudson M., Fernández-del C.C., Bauer T.W., Williams M., Warshaw A.L., Thayer S.P., Kelly K.A. (2011). Plectin-1 as a novel biomarker for pancreatic cancer. Clin. Cancer Res..

[12] Liu Y.H., Ho C.C., Cheng C.C., Pei R.J., Hsu Y.H., Yeh K.T., Tsai M.C., Lai Y.S. (2007). Pleomorphism of cancer cells with the expression of plectin and concept of filament bundles in human hepatocellular carcinoma. Res. Commun. Mol. Pathol. Pharmacol..

[13] Cheng C.C., Lai Y.C., Lai Y.S., Hsu Y.H., Chao W.T., Sia K.C., Tseng Y.H., Liu Y.H. (2015). Transient knockdown-mediated deficiency in plectin alters hepatocellular motility in association with activated FAK and Rac1-GTPase. Cancer Cell Int..

[14] Dumas V., Kanitakis J., Charvat S., Euvrard S., Faure M., Claudy A. (1999). Expression of basement membrane antigens and matrix metalloproteinases 2 and 9 in cutaneous basal and squamous cell carcinomas. Anticancer Res..

[15] Perez S.M., Brinton L.T., Kelly K.A. (2021). Plectin in Cancer: From Biomarker to Therapeutic Target. Cells.

[16] McInroy L., Määttä A. (2011). Plectin regulates invasiveness of SW480 colon carcinoma cells and is targeted to podosome-like adhesions in an isoform-specific manner. Exp. Cell Res..

[17] Winter L., Wiche G. (2013). The many faces of plectin and plectinopathies: Pathology and mechanisms. Acta Neuropathol..

[18] Castañón M.J., Wiche G. (2021). Identifying Plectin Isoform Functions through Animal Models. Cells.

[19] Chaudhari P.R., Vaidya M.M. (2015). Versatile hemidesmosomal linker proteins: Structure and function. Histol. Histopathol..

[20] Sonnenberg A., Liem R.K. (2007). Plakins in development and disease. Exp. Cell Res..

[21] Liu C.G., Maercker C., Castañon M.J., Hauptmann R., Wiche G. (1996). Human plectin: Organization of the gene, sequence analysis, and chromosome localization (8q24). Proc. Natl. Acad. Sci. USA.

[22] McLean W.H., Pulkkinen L., Smith F.J., Rugg E.L., Lane E.B., Bullrich F., Burgeson R.E., Amano S., Hudson D.L., Owaribe K. (1996). Loss of plectin causes epidermolysis bullosa with muscular dystrophy: cDNA cloning and genomic organization. Genes Dev..

[23] Fuchs P., Zörer M., Rezniczek G.A., Spazierer D., Oehler S., Castañón M.J., Hauptmann R., Wiche G. (1999). Unusual 5′ transcript complexity of plectin isoforms: Novel tissue-specific exons modulate actin binding activity. Hum. Mol. Genet..

[24] Rezniczek G.A., Abrahamsberg C., Fuchs P., Spazierer D., Wiche G. (2003). Plectin 5′-transcript diversity: Short alternative sequences determine stability of gene products, initiation of translation and subcellular localization of isoforms. Hum. Mol. Genet..

[25] Foisner R., Wiche G. (1987). Structure and hydrodynamic properties of plectin molecules. J. Mol. Biol..

[26] Wiche G., Becker B., Luber K., Weitzer G., Castañon M.J., Hauptmann R., Stratowa C., Stewart M. (1991). Cloning and sequencing of rat plectin indicates a 466-kD polypeptide chain with a three-domain structure based on a central alpha-helical coiled coil. J. Cell Biol..

[27] Hu L., Huang Z., Wu Z., Ali A., Qian A. (2018). Mammalian Plakins, Giant Cytolinkers: Versatile Biological Functions and Roles in Cancer. Int. J. Mol. Sci..

[28] Bouameur J.E., Favre B., Borradori L. (2014). Plakins, a versatile family of cytolinkers: Roles in skin integrity and in human diseases. J. Investig. Dermatol..

[29] Foisner R., Wiche G. (1991). Intermediate filament-associated proteins. Curr. Opin. Cell Biol..

[30] Starr D.A., Fridolfsson H.N. (2010). Interactions between nuclei and the cytoskeleton are mediated by SUN-KASH nuclear-envelope bridges. Annu. Rev. Cell Dev. Biol..

[31] De Pascalis C., Pérez-González C., Seetharaman S., Boëda B., Vianay B., Burute M., Leduc C., Borghi N., Trepat X., Etienne-Manneville S. (2018). Intermediate filaments control collective migration by restricting traction forces and sustaining cell-cell contacts. J. Cell Biol..

[32] de Pereda J.M., Lillo M.P., Sonnenberg A. (2009). Structural basis of the interaction between integrin alpha6beta4 and plectin at the hemidesmosomes. EMBO J..

[33] Wilhelmsen K., Litjens S.H., Kuikman I., Tshimbalanga N., Janssen H., van den Bout I., Raymond K., Sonnenberg A. (2005). Nesprin-3, a novel outer nuclear membrane protein, associates with the cytoskeletal linker protein plectin. J. Cell Biol..

[34] Ketema M., Wilhelmsen K., Kuikman I., Janssen H., Hodzic D., Sonnenberg A. (2007). Requirements for the localization of nesprin-3 at the nuclear envelope and its interaction with plectin. J. Cell Sci..

[35] García-Alvarez B., Bobkov A., Sonnenberg A., de Pereda J.M. (2003). Structural and functional analysis of the actin binding domain of plectin suggests alternative mechanisms for binding to F-actin and integrin beta4. Structure.

[36] Rezniczek G.A., Konieczny P., Nikolic B., Reipert S., Schneller D., Abrahamsberg C., Davies K.E., Winder S.J., Wiche G. (2007). Plectin 1f scaffolding at the sarcolemma of dystrophic (mdx) muscle fibers through multiple interactions with beta-dystroglycan. J. Cell Biol..

[37] Wang W., Zuidema A., Te Molder L., Nahidiazar L., Hoekman L., Schmidt T., Coppola S., Sonnenberg A. (2020). Hemidesmosomes modulate force generation via focal adhesions. J. Cell Biol..

[38] Koster J., van Wilpe S., Kuikman I., Litjens S.H., Sonnenberg A. (2004). Role of binding of plectin to the integrin beta4 subunit in the assembly of hemidesmosomes. Mol. Biol. Cell.

[39] Djinovic-Carugo K., Gautel M., Ylänne J., Young P. (2002). The spectrin repeat: A structural platform for cytoskeletal protein assemblies. FEBS Lett..

[40] Matsubara T., Yaginuma T., Addison W.N., Fujita Y., Watanabe K., Yoshioka I., Hikiji H., Maki K., Baron R., Kokabu S. (2020). Plectin stabilizes microtubules during osteoclastic bone resorption by acting as a scaffold for Src and Pyk2. Bone.

[41] Wenta T., Schmidt A., Zhang Q., Devarajan R., Singh P., Yang X., Ahtikoski A., Vaarala M., Wei G.H., Manninen A. (2022). Disassembly of α6β4-mediated hemidesmosomal adhesions promotes tumorigenesis in PTEN-negative prostate cancer by targeting plectin to focal adhesions. Oncogene.

[42] Frijns E., Kuikman I., Litjens S., Raspe M., Jalink K., Ports M., Wilhelmsen K., Sonnenberg A. (2012). Phosphorylation of threonine 1736 in the C-terminal tail of integrin β4 contributes to hemidesmosome disassembly. Mol. Biol. Cell.

[43] Jang T.H., Huang W.C., Tung S.L., Lin S.C., Chen P.M., Cho C.Y., Yang Y.Y., Yen T.C., Lo G.H., Chuang S.E. (2022). MicroRNA-485-5p targets keratin 17 to regulate oral cancer stemness and chemoresistance via the integrin/FAK/Src/ERK/β-catenin pathway. J. Biomed. Sci..

[44] Wiche G. (2021). Plectin-Mediated Intermediate Filament Functions: Why Isoforms Matter. Cells.

[45] Gundesli H., Kori M., Arga K.Y. (2023). The Versatility of Plectin in Cancer: A Pan-Cancer Analysis on Potential Diagnostic and Prognostic Impacts of Plectin Isoforms. Omics.

[46] Flores I.L., Kawahara R., Miguel M.C., Granato D.C., Domingues R.R., Macedo C.C., Carnielli C.M., Yokoo S., Rodrigues P.C., Monteiro B.V. (2016). EEF1D modulates proliferation and epithelial-mesenchymal transition in oral squamous cell carcinoma. Clin. Sci..

[47] Rodrigues P.C., Sawazaki-Calone I., Ervolino de Oliveira C., Soares Macedo C.C., Dourado M.R., Cervigne N.K., Miguel M.C., Ferreira do Carmo A., Lambert D.W., Graner E. (2017). Fascin promotes migration and invasion and is a prognostic marker for oral squamous cell carcinoma. Oncotarget.

[48] Rikardsen O.G., Magnussen S.N., Svineng G., Hadler-Olsen E., Uhlin-Hansen L., Steigen S.E. (2015). Plectin as a prognostic marker in non-metastatic oral squamous cell carcinoma. BMC Oral Health.

[49] Chaudhari P.R., Charles S.E., D’Souza Z.C., Vaidya M.M. (2017). Hemidesmosomal linker proteins regulate cell motility, invasion and tumorigenicity in oral squamous cell carcinoma derived cells. Exp. Cell Res..

[50] Schreurs O., Balta M.G., Karatsaidis A., Schenck K. (2020). Composition of hemidesmosomes in basal keratinocytes of normal buccal mucosa and oral lichen planus. Eur. J. Oral Sci..

[51] Dmello C., Sawant S., Alam H., Gangadaran P., Tiwari R., Dongre H., Rana N., Barve S., Costea D.E., Chaukar D. (2016). Vimentin-mediated regulation of cell motility through modulation of beta4 integrin protein levels in oral tumor derived cells. Int. J. Biochem. Cell Biol..

[52] Lee K.Y., Liu Y.H., Ho C.C., Pei R.J., Yeh K.T., Cheng C.C., Lai Y.S. (2004). An early evaluation of malignant tendency with plectin expression in human colorectal adenoma and adenocarcinoma. J. Med..

[53] Zheng S., Qin F., Yin J., Li D., Huang Y., Hu L., He L., Lv C., Li X., Li S. (2023). Role and mechanism of actin-related protein 2/3 complex signaling in cancer invasion and metastasis: A review. Medicine.

[54] Prosseda P.P., Alvarado J.A., Wang B., Kowal T.J., Ning K., Stamer W.D., Hu Y., Sun Y. (2020). Optogenetic stimulation of phosphoinositides reveals a critical role of primary cilia in eye pressure regulation. Sci. Adv..

[55] Jiu Y., Lehtimäki J., Tojkander S., Cheng F., Jäälinoja H., Liu X., Varjosalo M., Eriksson J.E., Lappalainen P. (2015). Bidirectional Interplay between Vimentin Intermediate Filaments and Contractile Actin Stress Fibers. Cell Rep..

[56] Pawar H., Kashyap M.K., Sahasrabuddhe N.A., Renuse S., Harsha H.C., Kumar P., Sharma J., Kandasamy K., Marimuthu A., Nair B. (2011). Quantitative tissue proteomics of esophageal squamous cell carcinoma for novel biomarker discovery. Cancer Biol. Ther..

[57] Knox J.D., Cress A.E., Clark V., Manriquez L., Affinito K.S., Dalkin B.L., Nagle R.B. (1994). Differential expression of extracellular matrix molecules and the alpha 6-integrins in the normal and neoplastic prostate. Am. J. Pathol..

[58] Davis T.L., Cress A.E., Dalkin B.L., Nagle R.B. (2001). Unique expression pattern of the alpha6beta4 integrin and laminin-5 in human prostate carcinoma. Prostate.

[59] Stanzani E., Pedrosa L., Bourmeau G., Anezo O., Noguera-Castells A., Esteve-Codina A., Passoni L., Matteoli M., de la Iglesia N., Seano G. (2021). Dual Role of Integrin Alpha-6 in Glioblastoma: Supporting Stemness in Proneural Stem-Like Cells While Inducing Radioresistance in Mesenchymal Stem-Like Cells. Cancers.

[60] Xu R., He S., Ma D., Liang R., Luo Q., Song G. (2022). Plectin Downregulation Inhibits Migration and Suppresses Epithelial Mesenchymal Transformation of Hepatocellular Carcinoma Cells via ERK1/2 Signaling. Int. J. Mol. Sci..

[61] Bausch D., Mino-Kenudson M., Fernández-Del Castillo C., Warshaw A.L., Kelly K.A., Thayer S.P. (2009). Plectin-1 is a biomarker of malignant pancreatic intraductal papillary mucinous neoplasms. J. Gastrointest. Surg..

[62] Moris M., Dawson D.W., Jiang J., Lewis J., Nassar A., Takeuchi K.K., Lay A.R., Zhai Q., Donahue T.R., Kelly K.A. (2016). Plectin-1 as a Biomarker of Malignant Progression in Intraductal Papillary Mucinous Neoplasms: A Multicenter Study. Pancreas.

[63] Mizuta K., Matsubara T., Goto A., Addison W.N., Nakatomi M., Matsuo K., Tada-Shigeyama Y., Yaginuma T., Honda H., Yoshioka I. (2022). Plectin promotes tumor formation by B16 mouse melanoma cells via regulation of Rous sarcoma oncogene activity. BMC Cancer.

[64] Sutoh Yoneyama M., Hatakeyama S., Habuchi T., Inoue T., Nakamura T., Funyu T., Wiche G., Ohyama C., Tsuboi S. (2014). Vimentin intermediate filament and plectin provide a scaffold for invadopodia, facilitating cancer cell invasion and extravasation for metastasis. Eur. J. Cell Biol..

[65] Reynolds F., Panneer N., Tutino C.M., Wu M., Skrabal W.R., Moskaluk C., Kelly K.A. (2011). A functional proteomic method for biomarker discovery. PLoS ONE.

[66] Mittal P., Klingler-Hoffmann M., Arentz G., Winderbaum L., Lokman N.A., Zhang C., Anderson L., Scurry J., Leung Y., Stewart C.J. (2016). Lymph node metastasis of primary endometrial cancers: Associated proteins revealed by MALDI imaging. Proteomics.

[67] Perez S.M., Dimastromatteo J., Landen C.N., Kelly K.A. (2021). A Novel Monoclonal Antibody Targeting Cancer-Specific Plectin Has Potent Antitumor Activity in Ovarian Cancer. Cells.

[68] Puiffe M.L., Le Page C., Filali-Mouhim A., Zietarska M., Ouellet V., Tonin P.N., Chevrette M., Provencher D.M., Mes-Masson A.M. (2007). Characterization of ovarian cancer ascites on cell invasion, proliferation, spheroid formation, and gene expression in an in vitro model of epithelial ovarian cancer. Neoplasia.

[69] Oto A., Eltorky M.A., Dave A., Ernst R.D., Chen K., Rampy B., Chaljub G., Nealon W. (2006). Mimicks of pancreatic malignancy in patients with chronic pancreatitis: Correlation of computed tomography imaging features with histopathologic findings. Curr. Probl. Diagn. Radiol..

[70] Wang C.I., Wang C.L., Wu Y.C., Feng H.P., Liu P.J., Chang Y.S., Yu J.S., Yu C.J. (2015). Quantitative proteomics reveals a novel role of karyopherin alpha 2 in cell migration through the regulation of vimentin-pErk protein complex levels in lung cancer. J. Proteome Res..

[71] Niwa T., Saito H., Imajoh-ohmi S., Kaminishi M., Seto Y., Miki Y., Nakanishi A. (2009). BRCA2 interacts with the cytoskeletal linker protein plectin to form a complex controlling centrosome localization. Cancer Sci..

[72] Cheng C.C., Liu Y.H., Ho C.C., Chao W.T., Pei R.J., Hsu Y.H., Yeh K.T., Ho L.C., Tsai M.C., Lai Y.S. (2008). The influence of plectin deficiency on stability of cytokeratin18 in hepatocellular carcinoma. J. Mol. Histol..

[73] Liu Y.H., Cheng C.C., Ho C.C., Chao W.T., Pei R.J., Hsu Y.H., Yeh K.T., Ho L.C., Tsai M.C., Lai Y.S. (2008). Degradation of plectin with modulation of cytokeratin 18 in human liver cells during staurosporine-induced apoptosis. In Vivo.

[74] Liu Y.H., Ho C.C., Cheng C.C., Chao W.T., Pei R.J., Hsu Y.H., Lai Y.S. (2011). Cytokeratin 18-mediated disorganization of intermediate filaments is induced by degradation of plectin in human liver cells. Biochem. Biophys. Res. Commun..

[75] Cheng C.C., Chao W.T., Liao C.C., Tseng Y.H., Lai Y.C., Lai Y.S., Hsu Y.H., Liu Y.H. (2018). Plectin deficiency in liver cancer cells promotes cell migration and sensitivity to sorafenib treatment. Cell Adhes. Migr..

[76] Raymond K., Kreft M., Song J.Y., Janssen H., Sonnenberg A. (2007). Dual Role of alpha6beta4 integrin in epidermal tumor growth: Tumor-suppressive versus tumor-promoting function. Mol. Biol. Cell.

[77] Ni Y., Wang X., Yin X., Li Y., Liu X., Wang H., Liu X., Zhang J., Gao H., Shi B. (2018). Plectin protects podocytes from adriamycin-induced apoptosis and F-actin cytoskeletal disruption through the integrin α6β4/FAK/p38 MAPK pathway. J. Cell. Mol. Med..

[78] Ridley A.J. (2006). Rho GTPases and actin dynamics in membrane protrusions and vesicle trafficking. Trends Cell Biol..

[79] Moch M., Windoffer R., Schwarz N., Pohl R., Omenzetter A., Schnakenberg U., Herb F., Chaisaowong K., Merhof D., Ramms L. (2016). Effects of Plectin Depletion on Keratin Network Dynamics and Organization. PLoS ONE.

[80] Bahadoran P., Perrin C., Aberdam D., Spadafora-Pisani A., Meneguzzi G., Ortonne J.P. (1997). Altered expression of the hemidesmosome-anchoring filament complex proteins in basal cell carcinoma: Possible role in the origin of peritumoral lacunae. Br. J. Dermatol..

[81] Dajee M., Lazarov M., Zhang J.Y., Cai T., Green C.L., Russell A.J., Marinkovich M.P., Tao S., Lin Q., Kubo Y. (2003). NF-kappaB blockade and oncogenic Ras trigger invasive human epidermal neoplasia. Nature.

[82] Caglar H.O., Biray Avci C. (2020). Alterations of cell cycle genes in cancer: Unmasking the role of cancer stem cells. Mol. Biol. Rep..

[83] Vermeulen K., Van Bockstaele D.R., Berneman Z.N. (2003). The cell cycle: A review of regulation, deregulation and therapeutic targets in cancer. Cell Prolif..

[84] Gaiko-Shcherbak A., Eschenbruch J., Kronenberg N.M., Teske M., Wolters B., Springer R., Gather M.C., Merkel R., Hoffmann B., Noetzel E. (2021). Cell Force-Driven Basement Membrane Disruption Fuels EGF- and Stiffness-Induced Invasive Cell Dissemination from Benign Breast Gland Acini. Int. J. Mol. Sci..

[85] Liotta L.A. (2016). Adhere, Degrade, and Move: The Three-Step Model of Invasion. Cancer Res..

[86] Winkler J., Abisoye-Ogunniyan A., Metcalf K.J., Werb Z. (2020). Concepts of extracellular matrix remodelling in tumour progression and metastasis. Nat. Commun..

[87] Lugano R., Ramachandran M., Dimberg A. (2020). Tumor angiogenesis: Causes, consequences, challenges and opportunities. Cell. Mol. Life Sci..

[88] Koivusalo S., Schmidt A., Manninen A., Wenta T. (2022). Regulation of Kinase Signaling Pathways by α6β4-Integrins and Plectin in Prostate Cancer. Cancers.

[89] Žugec M., Furlani B., Castañon M.J., Rituper B., Fischer I., Broggi G., Caltabiano R., Barbagallo G.M.V., Di Rosa M., Tibullo D. (2024). Plectin plays a role in the migration and volume regulation of astrocytes: A potential biomarker of glioblastoma. J. Biomed. Sci..

[90] Wu Y., Tang Y., Xie S., Zheng X., Zhang S., Mao J., Wang B., Hou Y., Hu L., Chai K. (2020). Chimeric peptide supramolecular nanoparticles for plectin-1 targeted miRNA-9 delivery in pancreatic cancer. Theranostics.

[91] Wesley T., Berzins S., Kannourakis G., Ahmed N. (2021). The attributes of plakins in cancer and disease: Perspectives on ovarian cancer progression, chemoresistance and recurrence. Cell Commun. Signal..

[92] Cheng C.C., Lai Y.C., Lai Y.S., Chao W.T., Tseng Y.H., Hsu Y.H., Chen Y.Y., Liu Y.H. (2016). Cell Pleomorphism and Cytoskeleton Disorganization in Human Liver Cancer. In Vivo.

[93] Jain R.K., Martin J.D., Stylianopoulos T. (2014). The role of mechanical forces in tumor growth and therapy. Annu. Rev. Biomed. Eng..

[94] Li N., Zhang X., Zhou J., Li W., Shu X., Wu Y., Long M. (2022). Multiscale biomechanics and mechanotransduction from liver fibrosis to cancer. Adv. Drug Deliv. Rev..

[95] Gargalionis A.N., Papavassiliou K.A., Basdra E.K., Papavassiliou A.G. (2022). mTOR Signaling Components in Tumor Mechanobiology. Int. J. Mol. Sci..

[96] Passi M., Zahler S. (2021). Mechano-Signaling Aspects of Hepatocellular Carcinoma. J. Cancer.

[97] Bregenzer M.E., Horst E.N., Mehta P., Novak C.M., Repetto T., Mehta G. (2019). The Role of Cancer Stem Cells and Mechanical Forces in Ovarian Cancer Metastasis. Cancers.

